# Radiative heat transfer between two carbon nanotubes

**DOI:** 10.1038/s41598-022-22138-8

**Published:** 2022-10-26

**Authors:** Igor S. Nefedov, Michael V. Davidovich, Olga E. Glukhova, Michael M. Slepchenkov, J. Miguel Rubi

**Affiliations:** 1 Department of Physics, Saratov State University, Astrakhanskaya street 83, Saratov, Russia 410012; 2 Laboratory of Biomedical Nanotechnology, I.M. Sechenov First Moscow State Medical University, Bolshaya Pirogovskaya Street 2-4, Moscow, Russia 119991; 3 Departament de Fisica de la Matèria Condensada, Universitat de Barcelona, Marti i Franquès 1, 08028 Barcelona, Spain

**Keywords:** Nanoscience and technology, Optics and photonics

## Abstract

We analyze the radiative heat transfer between two parallel and infinitely long carbon nanotubes (CNTs). The radiative heat exchange is due to the difference between the Poynting vectors generated by the fluctuating currents when the CNTs are at different temperatures. The radiated and absorbed Poynting vectors are expressed in terms of the correlations of the electromagnetic fields obtained from the Green’s function and the fluctuation-dissipation theorem for the current density. The analysis takes into account the scattering of the fields by the nanotubes. We show that the radiative heat transfer depends not only on the distance between nanotubes, but also on their chiralities and thus on their semiconducting or metallic nature, which would allow the design of nanostructures for optimal radiative heat exchange.

## Introduction

In their study of radiative heat transfer between two infinite parallel plates at different temperatures, Polder and van Hove^1^ showed that it is possible to exceed the value of the radiated energy, established by Planck’s law, when the distance between the plates is small enough for evanescent waves to contribute to the radiation, in the so-called near-field limit. This result was obtained under the assumption that thermal radiation is generated by the presence of fluctuations of electromagnetic fields, which are also responsible for some other phenomena such as thermal emission, van der Waals interactions, Casimir effect and van der Waals friction between two bodies^2^. Rytov’s fluctuational electrodynamics^3^ provides the theoretical framework to analyze the effect of fluctuations.

Radiative heat transfer arises from the difference between the Poynting vectors associated with the fluctuating electromagnetic fields that one object generates in the other when the two are at different temperatures. The Poynting vector is obtained from the distribution of electromagnetic fields in the objects, which are calculated using Green’s functions. This explains why radiative heat transfer depends on the geometry of the objects, as shown in^4,5^.

The important contribution of the evanescent waves in near-field radiative heat transfer has been reported for different basic configurations such as dipole-plate, dipole-dipole, sphere-sphere, sphere-plate, cylinder-cylinder, cone-plate, cone-cone and ring-ring^6–19^. The impact of radiative heat transfer on thermal technologies has been reviewed in^20^.

When the plates are made of graphene, near field heat transfer shows new behaviours that have been the subject of recent studies. It has been found to depend on doping and electron relaxation time, with maximum transfer observed at low doping and when the plasmons of the two graphene sheets are in resonance^21^. Also, the coupling of surface plasmon polaritons and surface phonon polaritons results in a colossal enhancement of the energy transmission coefficient and radiation spectra^22^. It has recently been shown that near field heat transfer can be significantly enhanced in multilayered graphene structures^23^.

In this article, we will analyze a new aspect of the near field radiative heat transfer between graphene structures. We will show that the near-field heat transfer between two CNTs depends significantly on their chiralities which dictate their metallic or semiconducting behaviour. It also depends on the relaxation time of the surface conductivity. The radiative heat transfer is higher when the nanotubes are metallic than when they are semiconducting and when one is metallic and the other semiconducting. Metallic CNTs are therefore optimal structures for heat exchange.

The article is organized as follows. In “The system of two carbon nanotubes” section, we present the model for the system of two CNTs. In “Electromagnetic fields” section, we compute the electromagnetic fields from the Green’s function formalism. In “Poynting vector and radiative heat transfer” section, we calculate the Poynting vectors and the radiative energy exchanged by the nanotubes at two different temperatures. The results obtained for the energy exchanged as a function of frequency, distance, chirality and relaxation time is computed in “Results and discussion” section. In the “Conclusions” section, we present our main conclusions.

**Figure 1:**
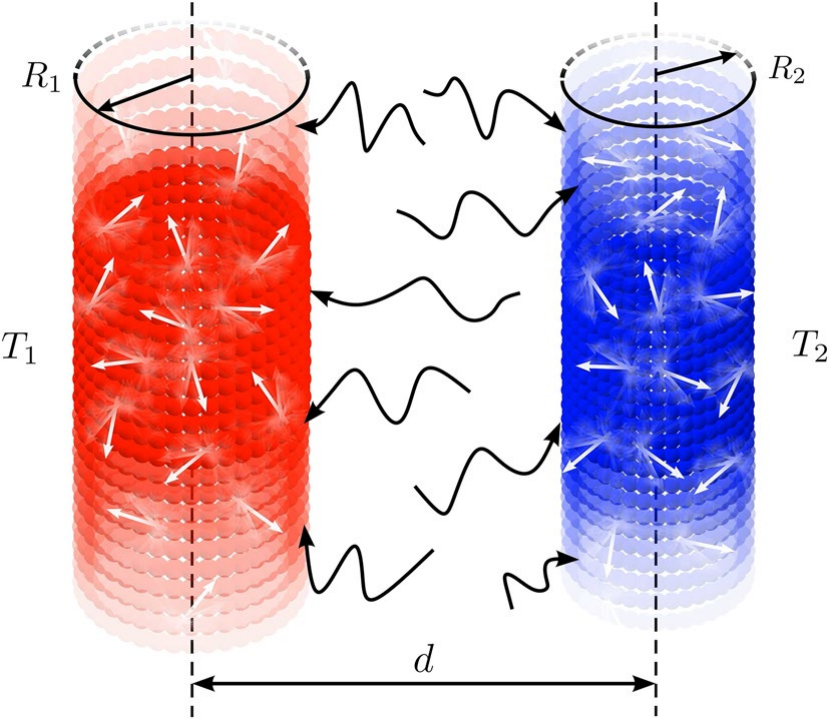
Radiative heat exchange between two parallel infinite-length carbon nanotubes of radii $R_{1}$ and $R_{2}$, separated by a distance of *d*. The nanotubes are at different temperatures, $T_{1}$ and $T_{2}$. The *z*-coordinate is directed along the nanotube axis while the *x*- and *y*-coordinates lie in the azimuthal plane.

## The system of two carbon nanotubes

We will study the radiative heat transfer between two parallel CNTs, represented in Fig. 1, having radii of a few nanometers and a length ranging up to centimeters^24^. Their electronic properties are determined by the way the graphene sheet is rolled-up. Two integers *m* and *n*, combining into a dual index (*m*, *n*), characterize different types of CNTs: $n=0$ for zigzag, $n=m$ for armchair, and $0<n\ne m$ for chiral ones. CNTs can manifest either metallic or semiconductor properties, depending on the chiral angle determined by indices *m* and *n*^25^. Armchair CNTs are always metallic (no energy bandgap), zigzag CNTs with $m=3q$, with *q* an integer, are also metallic. Other zigzag and chiral CNTs possess semiconductor properties. The radius of a nanotube can be expressed in terms of *m* and *n* as

1 $$\begin{aligned} R=\frac{\sqrt{3}}{2\pi }b\sqrt{m^2+mn+n^2}, \end{aligned}$$

where $b=0.142$ nm is the interatomic distance in graphene.

In this paper, we will consider zigzag CNTs whose radii are given by

2 $$\begin{aligned} R=\frac{\sqrt{3}bm}{2\pi }, \end{aligned}$$

where $b=0.142$ nm is the interatomic distance in single-wall CNTs. We will use the surface conductivity model, developed in^26^ which is based on effective boundary conditions and Green’s function methods and uses the $\pi$-electron tight-binding approximation. Formulas and parameter values are taken from^27^. According to this model, a CNT is considered as an impedance cylinder having a complex dynamic conductivity $\sigma (\omega )$ which includes the intraband and interband contributions

3 $$\begin{aligned} \sigma (\omega )=\sigma _{\mathrm{intra}}(\omega )+\sigma _{\mathrm{inter}}(\omega ). \end{aligned}$$

Explicit expressions for $\sigma _{\mathrm{intra}}(\omega )$ and $\sigma _{\mathrm{inter}}(\omega )$ are given in the Supplementary Material. This model is applicable to both metallic and semoconducting CNTs and is valid in any frequency range, including the visible one.

## Electromagnetic fields

In this Section, we will compute the electromagnetic (EM) field correlations for two parallel CNTs of infinite length having different radii and surface conductivities.

### Green’s functions

EM potentials $\mathbf{A}(\mathbf{r})$ can be expressed through dyadic Green’s function as^28^:

4 $$\begin{aligned} \mathbf{A}(\mathbf{r})=\int _V G(\mathbf{r},\mathbf{r}')\mathbf{J}(\mathbf{r}')\,d\mathbf{r}', \end{aligned}$$

where the vector $\mathbf{r}$ has components (*x*, *y*, *z*), $\mathbf{J}(\mathbf{r}')$ is the current density in a volume *V*, and $G(\mathbf{r},\mathbf{r}')$ is the scalar Green’s function. The time variation of the fields is given through the factor $\exp {(-i\omega t)}$. The dependence of all quantities on $\omega$ is omitted in Eq. (4) and henceforth.

The Fourier representation of the 3D Green’s function is

5 $$\begin{aligned} G(\mathbf{r},\mathbf{r}')=\frac{1}{(2\pi )^3}\int _{-\infty }^{\infty }\int _{-\infty }^{\infty }\int _{-\infty }^{\infty } \frac{\exp {(i[\alpha (x-x')+\beta (y-y')+\gamma (z-z')])}}{\alpha ^2+\beta ^2+\gamma ^2-k_0^2\epsilon _h}d\alpha d\beta d\gamma , \end{aligned}$$

where $\epsilon _h$ is the relative permittivity of the medium surrounding the CNTs. Integrating over $\alpha$ and using the residue method one obtains

6 $$\begin{aligned} G(\mathbf{r},\mathbf{r}')=\frac{1}{8\pi ^2}\int _{-\infty }^{\infty }\int _{-\infty }^{\infty }{\frac{\exp {(i[\beta (y-y') +\gamma (z-z')]-k_x|x-x'|])}}{k_x}}\,d\beta \,d\gamma , \end{aligned}$$

where $k_x=\sqrt{\gamma ^2+\beta ^2-k_0^2\epsilon _h}$, and if $\gamma ^2+\beta ^2<k_0^2\epsilon _h$ then the branch $k_x=-i\sqrt{k_0^2\epsilon _h-\gamma ^2-\beta ^2}$ is taken. The electric and magnetic fields can be expressed in terms of the Green function as

7 $$\begin{aligned} \mathbf{E}(\mathbf{r},\mathbf{r}')=\int _V\overline{\overline{G}}^e(\mathbf{r},\mathbf{r}')\mathbf{J}(\mathbf{r}')\,d\mathbf{r}', \end{aligned}$$

and

8 $$\begin{aligned} \mathbf{H}(\mathbf{r},\mathbf{r}')=\int _V\overline{\overline{G}}^h(\mathbf{r},\mathbf{r}')\mathbf{J}(\mathbf{r}')\,d\mathbf{r}', \end{aligned}$$

where $\overline{\overline{G}}^e(\mathbf{r},\mathbf{r}')$ and $\overline{\overline{G}}^h(\mathbf{r},\mathbf{r}')$ are the electric and magnetic dyadic Green’s functions, respectively.

The electric dyadic Green’s function is given by

9 $$\begin{aligned} \overline{\overline{G}}^e(\mathbf{r},\mathbf{r}')=\frac{i}{k_0}\eta (\nabla \nabla +k_0^2\epsilon _h\overline{\overline{I}})G(\mathbf{r},\mathbf{r}'), \end{aligned}$$

where $\eta =120\pi$ $\Omega$ (Ohm) is the wave impedance of vacuum. The magnetic dyadic Green’s function is

10 $$\begin{aligned} \overline{\overline{G}}^h(\mathbf{r},\mathbf{r}')=-{\hat{\epsilon }}_{\alpha \beta \gamma }\partial _{\gamma }G(\mathbf{r},\mathbf{r}'), \end{aligned}$$

where ${\hat{\epsilon }}_{\alpha \beta \gamma }$ is the Levi-Civita pseudotensor. The fields in Eqs. (7) and (8) contain both the fluctuation fields excited by current fluctuations and the scattered fields.

### EM fields

The radiative heat transfer between the CNTs can be obtained through the Poynting vector which is a bilinear form of the EM fields. Its average value is thus expressed in terms of correlations of the EM fields that can be computed from fluctuational electrodynamics^3^. We neglect the azimuthal currents in the CNTs because the axial component of the surface conductivity strongly dominates the azimuthal one due to quantum effects, hence $H_z=0$ and the radiative heat transfer in the *x*-direction is expressed only through $H_y$ and $E_z$.This assumption is justified when the diameters of the CNTs are small enough, of about 1 -1.5 nm smaller than the distance between them which is 10-20 nm The field components obtained from expressions (6)–(10) are given by

11 $$\begin{aligned} \begin{array}{l} E_z(\mathbf{r})=\frac{i}{8\pi ^2 k_0}\eta \int _V\int _{-\infty }^{\infty }\int _{-\infty }^{\infty } \frac{(k_0^2\epsilon _h-\gamma ^2) e^{i[\beta (y-y')+\gamma (z-z')]-k_x|x-x'|}}{k_x}\,J_z(\mathbf{r}') d\beta d\gamma \,d\mathbf{r}', \\ H_y(\mathbf{r})=-\frac{i}{8\pi ^2}\int _V\int _{-\infty }^{\infty }\int _{-\infty }^{\infty } e^{i[\beta (y-y')+\gamma (z-z')]-k_x|x-x'|}\, J_z(\mathbf{r}')\,d\beta d\gamma \,d\mathbf{r}' \end{array} \end{aligned}$$

where we have omitted the dependence of fields and currents on $\omega$. The electric and magnetic fields are expressed in terms of their Fourier transforms as

12 $$\begin{aligned} \begin{array}{lr} E_z(\mathbf{r})= \frac{1}{2\pi }\int _{-\infty }^{\infty } {{\tilde{E}}}_s(\mathbf{r}_{\perp })e^{i\gamma z}\, d\gamma ;&H_y(\mathbf{r}_{\perp })= \frac{1}{2\pi }\int _{-\infty }^{\infty } {{\tilde{H}}}_s(\mathbf{r}_{\perp })e^{i\gamma z}\, d\gamma , \end{array} \end{aligned}$$

where $\mathbf{r}_{\perp }=(x,y)$. Integration over the volumes of the nanotubes $V_1$ and $V_2$ thus reduces to integration over their cross-sectional areas $S_1$ and $S_2$.

Since the currents flow within shells of CNTs, we can replace the volume currents $J_z(\mathbf{r}'_{\perp })$ by the surface currents $J(\mathbf{r}'_{\perp })=\frac{2}{r}j_z(\mathbf{r}_{\perp }')$ (henceforth the subindex *z* will be omitted) and the integration over the CNT cross-sections by the integration over their contours, $L_1$ and $L_2$. We assume that the distribution of the surface current is homogeneous along the contour $L_m$ of the m-CNT, $m=1,2$, so the surface currents are $j_m(\mathbf{r}_{\perp })=j_m$, and thus denote

13 $$\begin{aligned} \begin{array}{lc} {{\mathcal {E}}}_m=\int _{L_m}{{\tilde{E}}}_z(l)\,dl,&{{\mathcal {H}}}_m=\int _{L_m}{{\tilde{H}}}_y(l)\,dl \end{array}\end{aligned}$$

Integrating Eqs. (11) and (12) over the coordinates $\mathbf{r}_{\perp }$ and $\mathbf{r}'_{\perp }$, we obtain:

14 $$\begin{aligned} \begin{array}{c} {{\mathcal {E}}}_1(\gamma )=-\frac{i}{8\pi ^2k_0}\eta (k_0^2\epsilon _h-\gamma ^2)\left\{ \frac{2}{R_1}\int _{-\infty }^{\infty }j_1(\beta ) \frac{1}{k_x}W_1 \,d\beta +\frac{2}{R_2}\int _{-\infty }^{\infty } j_2(\beta )W_{12}\frac{e^{-k_xd}}{k_x} \,d\beta \right\} \end{array} \end{aligned}$$

where

15 $$\begin{aligned} \begin{array}{c} W_1=\int _{L_1}\int _{L_1}e^{-k_x|x-x'|+i\beta (y-y')}\,dldl'= R_1^2\int _0^{2\pi }\int _0^{2\pi }e^{-k_xR_1|\sin {\phi }-\sin {\phi '}|+i\beta R_1(\cos {\phi }-\cos {\phi '})}\,d\phi d\phi '. \end{array} \end{aligned}$$

Similarly,

16 $$\begin{aligned} W_2=\int _{L_2}\int _{L_2}e^{-k_x|x-x'|+i\beta (y-y')}\,dldl'= R_2^2\int _0^{2\pi }\int _0^{2\pi }e^{-k_xR_2|\sin {\phi }-\sin {\phi '}|+i\beta R_2(\cos {\phi }-\cos {\phi '})}\,d\phi d\phi '. \end{aligned}$$

The integrals in $W_1$ and $W_2$ can be calculated numerically or replaced by simple expressions using the mean value theorem:

17 $$\begin{aligned} \begin{array}{cc} W_1=(2\pi R_1)^2,&W_2=(2\pi R_2)^2. \end{array}\end{aligned}$$

We now calculate the term

18 $$\begin{aligned} W_{12}=\int _{L_1}\int _{L_2}e^{-k_x|x-x'|+i\beta (y-y')}. \end{aligned}$$

Here $x'>x$ because this point belongs to the second CNT, so $|x-x'|=x'-x$. Therefore, one can write

19 $$\begin{aligned} W_{12}&=\int _{L_1}\int _{L_2}e^{-k_x|x-x'|+i\beta (y-y')}\,dldl'=\int _{L_1}e^{k_xx+i\beta y}\,dxdy \int _{L_2}e^{-k_xx'-i\beta y'}\,dx'dy'\nonumber \\&=R_1\int _0^{2\pi }e^{R_1(k_x\sin {\phi }+i\beta \cos {\phi })}\,d\phi R_2\int _0^{2\pi }e^{-R_2(k_x\sin {\phi '}+i\beta \cos {\phi '})}\,d\phi '\nonumber \\&=(2\pi )^2R_1R_2I_0(R_1\sqrt{\gamma ^2-k_0^2\epsilon _h})I_0(R_2\sqrt{\gamma ^2-k_0^2\epsilon _h})\equiv (2\pi )^2R_1R_2U, \end{aligned}$$

where $I_0(x)$ is the zero-order modified Bessel function. If $\gamma ^2<k_0^2\epsilon _h$, then $I_0(R_n\sqrt{\gamma ^2-k_0^2\epsilon _h}),\;m=1,2$ is replaced by $J_0(R_m\sqrt{k_0^2\epsilon _h-\gamma ^2})$, where $J_0(x)$ is the Bessel function. Then expression (14) can be written as:

20 $$\begin{aligned} {{\mathcal {E}}}_1(\gamma )=i\eta \frac{R_1}{k_0}(k_0^2\epsilon _h-\gamma ^2)\left[ \int _{-\infty }^{\infty } j_1(\beta )\frac{1}{k_x} \,d\beta +\int _{-\infty }^{\infty } j_2(\beta ) U\frac{1}{k_x}e^{-k_xd}\,d\beta \right] .\end{aligned}$$

For the second CNT, one can write:

21 $$\begin{aligned} {{\mathcal {E}}}_2(\gamma )=i\eta \frac{R_2}{k_0}(k_0^2\epsilon _h-\gamma ^2)\left[ \int _{-\infty }^{\infty } j_1(\beta )U\frac{1}{k_x}e^{-k_xd} \,d\beta +\int _{-\infty }^{\infty } j_2(\beta ) \frac{1}{k_x}\,d\beta \right] . \end{aligned}$$

The magnetic field components are

22 $$\begin{aligned} \begin{array}{lc} {{\mathcal {H}}}_1(\gamma )=-iR_1\left[ \int _{-\infty }^{\infty } j_1(\beta ) \,d\beta +\int _{-\infty }^{\infty } j_2(\beta ) Ue^{-k_xd}\,d\beta \right] \\ {{\mathcal {H}}}_2(\gamma )=-iR_2\left[ \int _{-\infty }^{\infty } j_1(\beta )Ue^{-k_xd} \,d\beta +\int _{-\infty }^{\infty } j_2(\beta ) \,d\beta \right] . \end{array}\end{aligned}$$

Thus, the average Poynting vector on the surface of the 2nd CNT, induced by the current in the 1st CNT, is expressed in terms of the current correlations as:

23 $$\begin{aligned} \langle {{\mathcal {E}}}_2{{\mathcal {H}}}_2^*\rangle (\gamma )= 4\eta \frac{R_2^2}{k_0}(k_0^2\epsilon _h-\gamma ^2)U^2\int _0^{\infty }\int _0^{\infty } \frac{e^{-(k_x+k_x')d}}{k_x}\langle j_1(\beta ) j_1(\beta ')\rangle \,d\beta \,d\beta '. \end{aligned}$$

## Poynting vector and radiative heat transfer

To obtain the Poynting vector from the result obtained in the previous equation, we will calculate the current and its correlations.

### Calculation of the current

The electromagnetic fields are solutions of the Lippmann-Schwinger integral equations^29^:

24 $$\begin{aligned} \begin{array}{c} E_z(\mathbf{r})=E_z^0(\mathbf{r}) +\int _V G^e_{zz}(\mathbf{r},\mathbf{r}')J_z(\mathbf{r}')\,d\mathbf{r}', \\ H_y(\mathbf{r})=H_y^0(\mathbf{r}) +\int _V G^h_{yz}(\mathbf{r},\mathbf{r}')J_z(\mathbf{r}')\,d\mathbf{r}', \end{array} \end{aligned}$$

where $G^e_{zz}$ and $G^h_{yz}$ are the *zz* and *yz* components of the electric and magnetic Green’s tensors, respectively. The field components $E_z(\mathbf{r}),\;H_y(\mathbf{r})$, and the current $J_z(\mathbf{r})$ consist of contributions due to EM fluctuations and to the scattered (diffraction) fields, that will be respectively denoted by the indexes 0 and *s*. We then have: $E_z(\mathbf{r})=E_z^0(\mathbf{r})+E_z^s(\mathbf{r})$, $H_y(\mathbf{r})=H_y^0(\mathbf{r})+H_y^s(\mathbf{r})$, and $J_z(\mathbf{r})=J_z^0(\mathbf{r})+J_z^s(\mathbf{r})$. The total fields can be found by solving Eq. (24) by the iteration method of the multiple scattering approach^30,31^. In the zero-order approximation, which was used in^32^ to calculate the Casimir force between two CNTs, $E_q(\mathbf{r})=E_q^0(\mathbf{r}),\ H_q(\mathbf{r})=H_q^0(\mathbf{r})$. Here we will find an exact solution for the scattered currents and substitute it into Eqs. (24). The scattered electric field on the CNT fulfills the impedance condition $j_m^s=\sigma _m E^s_z$ ($m=1,2$), where $\sigma _m$ is the surface conductivity of the *m*-th CNT. Then, imposing impedance boundary conditions at the surfaces of both CNTs, and using the Fourier transforms of the fields given in Eqs. (20),(21), one can write the system of linear equations for the Fourier transforms in $\gamma$ and $\beta$ of the currents:

25 $$\begin{aligned} \begin{array}{c} j_1^s(\beta )/\sigma _1=\Gamma _{11}(\beta )(j_0(\beta )+j_1^s(\beta ))+ \Gamma _{12}(\beta )j_2^s(\beta ), \\ j_2^s(\beta )/\sigma _2=\Gamma _{21}(\beta )(j_0(\beta )+j_1^s(\beta ))+ \Gamma _{22}(\beta )j_2^s(\beta ), \end{array}\end{aligned}$$

where

26 $$\begin{aligned} \begin{array}{cccc} \Gamma _{11}(\beta )=\eta \frac{k_0\epsilon _h-\gamma ^2}{2\pi k_0k_x},&\Gamma _{12}(\beta )=\Gamma _{11}(\beta )Ue^{-k_xd},&\Gamma _{21}(\beta )=\Gamma _{12}(\beta ),&\Gamma _{22}(\beta )=\Gamma _{11}(\beta ). \end{array}\end{aligned}$$

Here the dependence on $\gamma$ has been omitted and it has been assumed that only scattered currents flow in the 2nd CNT. Then we can express the scattered currents through fluctuating current of the 1-st CNT:

27 $$\begin{aligned} j_1^s(\beta )=j_0\frac{\Gamma _{11}Q_2-\Gamma _{12}\Gamma _{21}}{Q_1Q_2-\Gamma _{12}\Gamma _{21}}\equiv j_0D_1, \end{aligned}$$

where

28 $$\begin{aligned} \begin{array}{cc} Q_1=\frac{1}{\sigma _1}-\Gamma _{11},&Q_2=\frac{1}{\sigma _2}-\Gamma _{22}. \end{array}\end{aligned}$$

The total current in the 1st CNT then reads

29 $$\begin{aligned} j_1(\beta )=j_0(\beta )(1+D_1). \end{aligned}$$

### Poynting vector and radiative heat transfer

Substituting Eq. (29) for the full current into the fluctuation-dissipation theorem^33^, expressing the current fluctuations via the real part of surface conductivity^32^, we obtain

30 $$\begin{aligned} \langle j_1(\omega ,\gamma ,\beta )j_1(\omega ',\gamma ',\beta ')\rangle =\frac{(1+D)^2}{\pi }\sigma '_{1}\Theta (\omega ,T) \delta (\omega -\omega ')\delta (\gamma -\gamma ')\delta (\beta -\beta '), \end{aligned}$$

where

31 $$\begin{aligned} \Theta (\omega ,T)=\frac{\hbar \omega }{\exp {\left( \frac{\hbar \omega }{k_BT}-1\right) }}. \end{aligned}$$

is Planck’s distribution and $\sigma '_1=\sigma _{1zz}'$ is the real part of the *zz*-component of the surface conductivity tensor of the first CNT. Substituting (30) into (23), one obtains the expression for the Fourier components of the Poynting vector radiated by the first CNT at temperature $T_{1}$ and absorbed by the second

32 $$\begin{aligned} \langle {{\mathcal {E}}}_2{{\mathcal {H}}}_2^*\rangle (\omega ,\gamma ,T_1)= 4\eta \frac{R_2^2}{\pi k_0}(k_0^2\epsilon _h-\gamma ^2)\sigma _1'U^2\Theta (\omega ,T_1)\int _0^{\infty }\int _0^{\infty } (1+D_1)^2\frac{e^{-(k_x+k_x')d}}{k_x}\rangle \,d\beta \,d\beta '. \end{aligned}$$

Similarly, the Poynting vector radiated by the second CNT at $T_2$, and absorbed by the first CNT is

33 $$\begin{aligned} \langle {{\mathcal {E}}}_1{{\mathcal {H}}}_1^*\rangle (\omega ,\gamma ,T_2)= 4\eta \frac{R_1^2}{\pi k_0}(k_0^2\epsilon _h-\gamma ^2)\sigma _2'U^2\Theta (\omega ,T_2)\int _0^{\infty }\int _0^{\infty } (1+D_2)^2\frac{e^{-(k_x+k_x')d}}{k_x}\rangle \,d\beta \,d\beta ', \end{aligned}$$

where $D_2$ is obtained from $D_1$ by replacing $\sigma _1'\rightarrow \sigma _2'$, $\sigma _2'\rightarrow \sigma _1'$. Due to the parity of the integrands, we have replaced the lower integration limit $-\infty$ of the integrals over $\beta$ and $\beta '$ by zero and multiplied the integrals by a factor of two.

The total radiative heat transfer per unit length between both CNTs is thus given by

34 $$\begin{aligned} S_x(T_1,T_2)=\int _0^{\infty }S_{\omega }\,d\omega =4\int _{0}^{\infty }\,d\omega \int _0^{\infty }\left[ \langle {{\mathcal {E}}}_2{{\mathcal {H}}}_2^*\rangle (\omega ,\gamma ,T_1)-\langle {{\mathcal {E}}}_1 {{\mathcal {H}}}_1^*\rangle (\omega ,\gamma ,T_2)\right] \,d\gamma \,d\omega . \end{aligned}$$

Its behavior as a function of frequency and chirality will be analyzed in the next Section.

## Results and discussion

In this Section, we will present the most salient features of the near-field radiative heat transfer between different types of CNTs: metallic-metallic, metallic-semiconducting, and semiconducting-semiconducting. Metallic CNTs are of zigzag or armchair types whereas semiconducting CNTs are of zigzag type. The temperature of the first CNT is assumed to be 307 K which corresponds to the black-body thermal emission spectrum at about 18 THz. For simplicity, the temperature of the second CNT is taken as zero. The distance between both CNTs is 10 nm, and the relaxation time is $10^{-13}$ s for both CNTs, in the three cases considered.

**Figure 2:**
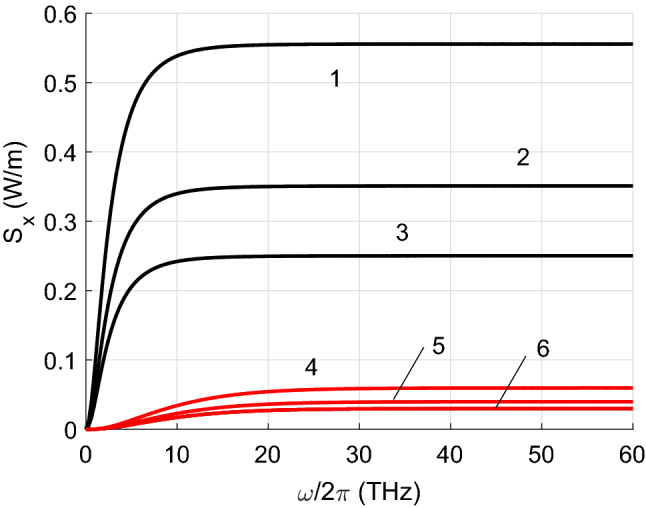
Radiative heat transfer per unit length versus frequency for $m_2=12$. Red curves relate to calculations with neglect of the scattering (lines 4,5,6). Black curves (1,2,3) show results obtained taking into account the scattering. $m_1=6$ (lines 1 and 4); $m_1=9$ (2 and 5); $m_1=12$ (3 and 6).

**Figure 3:**
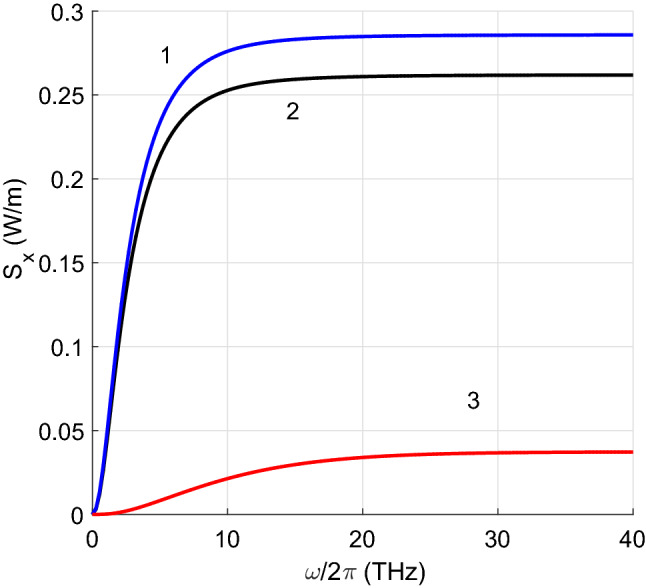
Radiative heat transfer per unit length $S_x$ versus frequency calculated for zigzag CNTs with $m_1=m_2=15$ (blue curve 1) and armchair CNTs with $m_1=m_2=9$ (black curve). The red curve is obtained for the case of absence of scattering.

**Figure 4:**
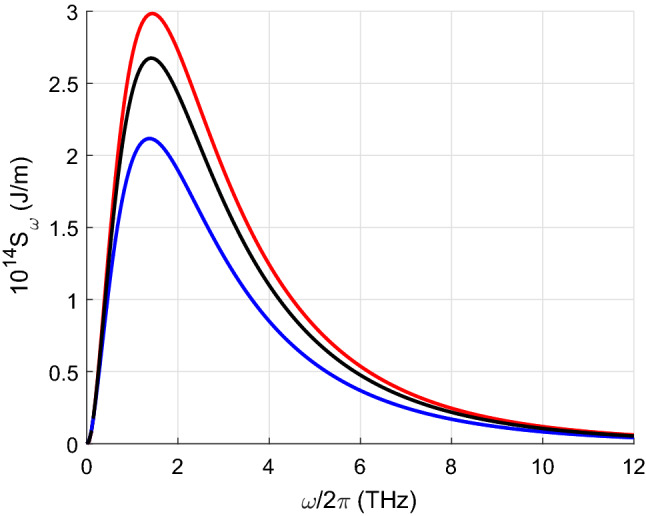
Spectral dependence of the radiative heat transfer $S_{\omega }(\omega /2\pi )$ per unit length calculated for armchair CNTs with $m_1=n_1=m_2=n_2=7$ (blue curve), $m_1=m_2=10$ (black curve), and $m_1=m_2=12$ (red curve).

**Figure 5:**
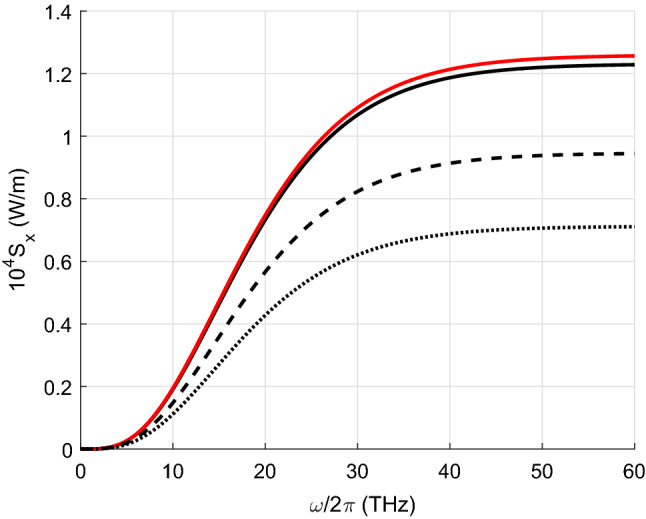
Radiative heat transfer per unit length $S_x$ versus frequency for $m_2=12$. The solid black curve corresponds to $m_1=13$ whereas the red one is obtained when scattering is not considered. The dashed line is obtained for $m_1=10$, and the dotted line for $m_1=8$.

Figures 2, 3, 4 and 5 show the results of the numerical integration over frequency of the Poynting vector, emitted by the first CNT. The integration stops when the integral converges which allow us to identify the frequency range contributing to the total thermal energy flow. In addition, we present results for the Poynting vector when scattering is considered or not. In the figure we plot the ratio between the total radiative heat transfer $S_x$ and that obtained only by propagating waves $S_0$. It can be seen that the evanescent waves are dominant in the heat transfer.

**Figure 6:**
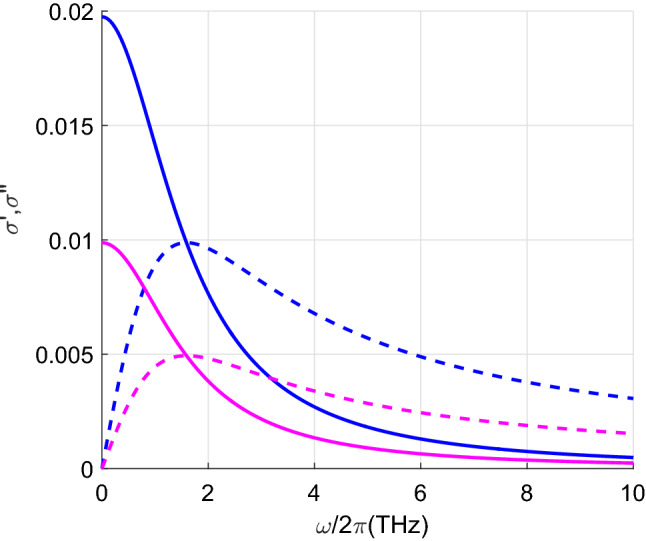
Real and imaginary parts of the surface conductivity, $\sigma _1'$ (solid lines) and $\sigma _1''$ (dashed lines), versus frequency. Blue lines stand for $m_1=6$ and magenta lines for $m_1=12$.

Figure 2 illustrates the importance of scattering in metallic CNTs. For $m_1=6$, the Poynting vector obtained when the scattering is considered is more than seven times the value obtained when scattering is not taken into account. This result indicates the existence of multiple scattering between the highly conductive metallic CNTs. This difference decreases rapidly with increasing distance *d*. For example, if $d=20$ nm, neglecting the scattering leads to an underestimation of the total heat transfer by about four times. It is observed that the lower the index $m_1$ (the smaller the diameter of the CNT) the higher the total radiative heat transfer. This fact can be explained by the fact that the real and imaginary parts of $\sigma _1$ are larger for CNTs with smaller indices $m_1$, see Fig. 6. Both $\sigma _1'$ and $\sigma _1''$ do matter since $\sigma _1''$ influence on the scattering. Note that for semiconducting CNTs both $\sigma '$ and $\sigma ''$ are two orders of magnitud smaller than for metallic CNTs^26^. It is remarkable that the radiative heat flow carried only by the propagating waves is ten orders of magnitude smaller than the total flux.

In Fig. 3, we compare the radiative heat flows between two metallic zigzag and two armchair CNTs. The *m* indices for both types of CNTs are taken in such a way that the radii were similar: $R=0.587$ nm for zigzag CNTs and $R=0.610$ nm for armchair CNTs. Comparison of the properties of different types of CNTs with equal radii is difficult as they take discrete values. The radiative heat transfer between zigzag CNTs is slightly higher. If scattering is ignored, the curve (in red) is close to the others.

Figure 4 illustrates the spectral dependence of the radiative heat transfer calculated for armchair CNTs with different radii determined by Eq. (1) with $m_1=n_1=m_2=n_2$. Figure 5 depicts the radiative heat exchange between CNTs when the first one has semiconducting properties and the second one metallic. We assume that the chiral index of the second CNT is $m_2=12$. There are two significant differences with respect to the previous case. First, the total heat transfer is almost four orders of magnitude lower than in the case of two metallic CNTs. This result is due to the lower conductivity of semiconducting CNTs. Secondly, the effect of scattering is very low, so we present the result without taking scattering into account only for $m_1=13$. The fields radiated by the first semiconductor CNT are scattered by the second metallic CNT, but do not excite any significant current in the first CNT, except for that induced by thermal fluctuations. For the same reason, the radiative heat transport between two semiconducting CNTs is much weaker than when both are metallic and similar to that when one is semiconducting and the other metallic as discussed above. Thirdly, unlike in the case of two metallic CNTs, the larger the index $m_1$ and consequently the radius of the CNT, the larger the total heat flux.

Calculation of the Poynting vectors have been carried out by using the values of the real and imaginary parts of $\sigma _1$ represented in Fig. 6. Both parts contribute to the result since $\sigma '$ is included in the fluctuation-dissipation theorem and $\sigma ''$ influences the scattering. For semiconducting CNTs $\sigma '$ and $\sigma ''$ are two orders of magnitude smaller than for metallic CNTs. We can also see in the figure that for CNTs with a lower index $m_1$ the real and imaginary parts of $\sigma _1$ are larger.

**Figure 7:**
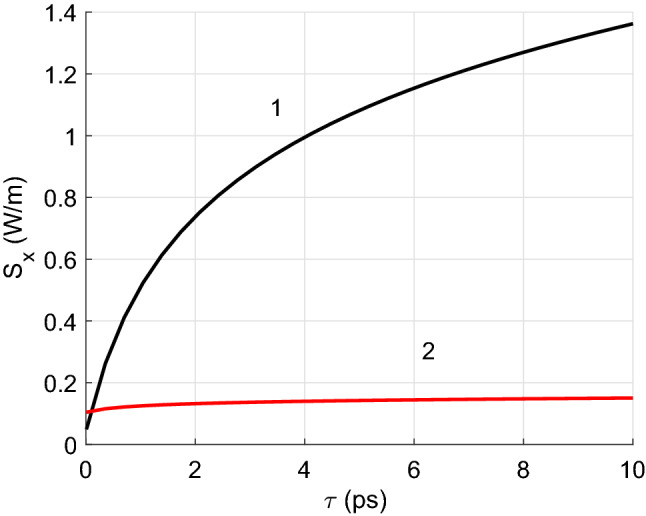
Radiative heat transfer per unit length versus relaxation time (black curve), with $\tau _1=\tau _2$, for metallic zigzag CNTs with $m_1=m_2=15$, and $d=20$ nm. The red line shows the result obtained when scattering is neglected.

Figure 7 displays the dependence of the radiated heat flux on the relaxation time $\tau$ responsible for the losses. This parameter can be estimated from the interaction of electrons with longitudinal acoustic phonons^34^. Calculations have been implemented for larger distance between CNTs than in previous cases, $d=20$ nm. How it depends on frequency is still a matter of debate. At low frequencies of about a few terahertz, it is usually taken as $10^{-12}$ to $10^{-13}$ s (see Refs.^26,35,36^). At higher infrared frequencies (below optical transitions) the value is $\tau = 10^{-13}$ to $10^{-14}$ s (see Ref.^37^). Due to the difficulty in determining this parameter, we have studied how the radiative heat transfer and the corresponding losses in metallic CNTs depend on it. The figure shows that the heat transfer increases with relaxation time. The results start from $\tau =0.01$ ps. The red curve 2 corresponds to the case of absence of scattering by the CNTs.

**Figure 8:**
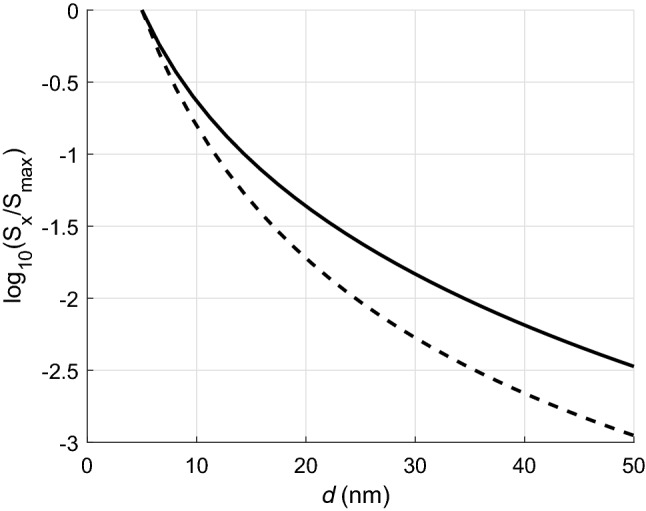
Normalized radiative heat transfer per unit length versus distance between CNTs (in nanometers), plotted in a logarithmic scale, computed for $m=15$, $T=307$ K, $\tau =\tau _1=\tau _2=10^{-13}$ s (solid curve), and $\tau =10^{-14}$ s (dashed curve).

Finally, Fig. 8 illustrates how the radiative flow decreases with increasing the distance between the nanotubes. The results are normalised to the maximum values of the radiative flux which are 1.221 W/m for $\tau =10^{-13}$ s, and 0.311 W/m for $\tau =10^{-14}$ s, respectively. They are given on a logarithmic scale due to the rapid drop in radiative heat transfer.

## Conclusions

In this article, we have studied the radiative heat transfer between two CNTs. Carrying out this study for these particular structures is important, as they are often found in many condensed matter, electronic, and biological applications. We have shown that the heat transfer is dependent on the distance between the nanotubes, their chiralities, their nature: metallic or semiconducting, and the relaxation time of the surface conductivity.

In the calculation, we have considered the CNTs to be parallel and infinitely long and have therefore presented the radiative heat transfer per unit length. We have assumed that the azimuthal currents on the CNT surfaces are negligible because the azimuthal component of the surface conductivity is much larger than the axial one, along the *z*-coordinate. Furthermore, we have considered a homogeneous distribution of the azimuthal surface current. Our analysis takes into account the fields scattered by the nanotubes. We have shown that the scattering is only important if both CNTs are metallic, with the corresponding conductivity value. We have found that the total radiative heat transfer between metallic CNTs is four orders of magnitude higher than in the cases of semiconductor and semiconductor-metallic CNTs.

## Supplementary Information

Supplementary Information.

## Data Availability

The data that support the findings of this study are available from the corresponding authors upon reasonable request.
