# Supplementary material for: Radiative heat transfer between two carbon nanotubes
Source: Sci Rep. 2022 Oct 26;12:17930. doi: 10.1038/s41598-022-22138-8 (PMC9606312; doi:10.1038/s41598-022-22138-8)
Supplement: Supplementary file 1 — Supplementary Information. [file 41598_2022_22138_MOESM1_ESM.pdf]

Supplementary Material

To compute the Poynting vector, we need to know the expression of the surface conductivity. This quantity consists of intraband and interband contributions given by the expressions[1,2]

$$\sigma_{\rm intra}(\omega)=\frac{ie^2}{2\pi^2 r\hbar(\omega+i/\tau_1)}\sum_{s=1}^{m}\int_{-a}^{a}\frac{\partial\mathcal{W}_c(p_z,s)}{\partial p_z}\frac{\partial\rho^{eq}(p_z,s)}{\partial p_z}\,dp_z, \tag{1}$$

$$\sigma_{\rm inter}(\omega)=\frac{ie^2}{\pi^2 r\hbar^2}\sum_{s=1}^{m}\int_{-a}^{a}\frac{\omega_{cv}(p_z,s)R_{cv}(p_z,s)\rho^{eq}(p_z,s)(\omega+i/\tau_2)}{\omega_{cv}^2(p_z,s)-(\omega+i/\tau_2)^2}\,dp_z. \tag{2}$$

where e is the electron charge. Here, the phenomenological parameters τ_1 and τ_2 are relaxation times and the integration is performed over the axial component of the momentum p_z within the first Brillouin zone, $a=\frac{2\pi\hbar}{\sqrt{3}b}$. The expressions of the different functions in Eqs. (1) and (2) are given below.

The frequency of the electron interband transitions ω_{cv} and the equilibrium inversion ρ^{eq}, related to the dispersion law of π electrons are given by

$$\omega_{cv}(p_z,s)=\frac{\mathcal{W}_c(p_z,s)-\mathcal{W}_v(p_z,s)}{\hbar}, \tag{3}$$

$$\rho^{eq}=F[\mathcal{W}_c(p_z,s)]-F[\mathcal{W}_v(p_z,s)], \tag{4}$$

where

$$F(\mathcal{W})=\left[1+\exp\left(\frac{\mathcal{W}}{k_BT}\right)\right]^{-1} \tag{5}$$

is the Fermi distribution, k_B the Boltzmann constant. In the framework of the tight-binding approximation**?** , for zigzag $(m,0)$ CNTs one has:

$$\mathcal{W}_{c,v}(p_z,s)=\pm\Gamma_0\left[1+4\cos\left(\frac{3bp_z}{2\hbar}\right)\cos\left(\frac{\pi s}{m}\right)+4\cos^2\left(\frac{\pi s}{m}\right)\right]^{1/2}, \tag{6}$$

$$R_{c,v}(p_z,s)=-\frac{b\Gamma_0^2}{2\mathcal{E}_c^2(p_z,s)}\left[1+\cos\left(\frac{3bp_z}{2\hbar}\right)\cos\left(\frac{\pi s}{m}\right)-2\cos^2\left(\frac{\pi s}{m}\right)\right], \tag{7}$$

where $\Gamma_0=2.7$ eV is the overlapping integral.

$$R_{cn}=\tfrac{\sqrt{3}bm}{2\pi},\ \ a=\tfrac{2\pi\hbar}{3b}. \tag{8}$$

For armchair (m,m) CNTs these functions are:

$$\mathcal{W}_{c,v}(p_z,s)=\pm\Gamma_0\left[1+4\cos\left(\frac{\sqrt{3}bp_z}{2\hbar}\right)\cos\left(\frac{\pi s}{m}\right)+4\cos^2\left(\frac{\sqrt{3}bp_z}{2\hbar}\right)\right]^{1/2}, \tag{9}$$

$$R_{c,v}(p_z,s)=-\frac{\sqrt{3}b\Gamma_0^2}{2\mathcal{E}_c^2(p_z,s)}\sin\left(\frac{\sqrt{3}bp_z}{2\hbar}\right)\sin\left(\frac{\pi s}{m}\right), \tag{10}$$

$$R_{cn}=\tfrac{3bm}{2\pi},\ \ a=\tfrac{2\pi\hbar}{\sqrt{3}b}. \tag{11}$$

[1] G.Y. Slepyan, S. A. Maksimenko, A. Lakhtakia, O. Yevtushenko and A.V. Gusakov, Phys. Rev. B **60**, 17136 (1999).
[2] A.M. Nemilentsau, J. Nanophotonics, **5**, 050401, (2011).
